# Assessment improved of cognitive impairment with artificial intelligence in the user‐web‐mobile application

**DOI:** 10.1002/alz.090025

**Published:** 2025-01-09

**Authors:** Arturo Del Bosque‐Díaz De León, Mireya Saraí García‐Vázquez, Alejandro Álvaro Ramírez‐Acosta, Sara Gloria Aguilar‐Navarro, Alberto Jose Mimenza

**Affiliations:** ^1^ Instituto Politécnico Nacional, Zacatecas, ZT Mexico; ^2^ Instituto Politécnico Nacional, CITEDI, Tijuana, BJ Mexico; ^3^ MIRAL R&D&I Multimedia, San Diego, CA USA; ^4^ Instituto Nacional de Ciencias Médicas y Nutrición Salvador Zubirán, México, DF Mexico

## Abstract

**Background:**

The World Health Organization forecasts a population of 2,000 million people over 60 years by the year 2050, with 7% of this population suffering from dementia. Making a constant clinical‐technological evaluation of older adults allows early detection of the disease and provides a better quality of life for the patient. In this sense, the research and development of innovative technological systems for the early detection of the disease, its monitoring and management of the growing number of patients with cognitive diseases has increased in recent years, integrating data collection and its automatic processing based on geriatric metrics into these systems using artificial intelligence (AI) methods.

**Method:**

This research presents an interactive web platform that allows users with any intelligent device with internet access, to remotely perform an automated assessment of the Montreal Cognitive Assessment (MoCA) test. We use AI and neural network methods for binary and multiclass classification to obtain assessment scores according to geriatric metrics. The application provides an automated evaluation of the MoCA test, which can then be validated remotely by a mental health specialist.

**Result:**

The tests performed show a correct correspondence in the handling of the information and results of each MoCA item with respect to the database. For the test database evaluated with the application, results are obtained with 100% accuracy and equal to the evaluations performed by specialists.

**Conclusion:**

This automated assessment provides great help to the medical specialist in the process of detection and evaluation of cognitive impairment, significantly improving the quality of healthcare. The management and organization for the follow‐up of the patient’s cognitive impairment is done through the information of the tests performed, their evaluation and the clinical history of each patient. This information is consulted and managed by the doctor for the patient’s follow‐up; the caregiver/family member has access to tests performed, their evaluation and all the follow‐up that the doctor gives to the patient.

The interface was developed thinking in the elderly. It is intuitive, with the relevant information and graphic elements, the procedure for using the application is explained step‐by‐step, the colors used are comfortable for eye care.

